# Natural surfactant mediated bioremediation approaches for contaminated soil

**DOI:** 10.1039/d3ra05062a

**Published:** 2023-10-18

**Authors:** Pintu Sar, Sandip Kundu, Aniruddha Ghosh, Bidyut Saha

**Affiliations:** a Department of Chemistry, The University of Burdwan Golapbag Burdwan 713104 WB India pintusar1@gmail.com aniruddhachem27@gmail.com bsaha@chem.buruniv.ac.in; b Department of Chemical Sciences, Indian Institute of Science Education and Research Kolkata Mohanpur – 741246 West Bengal India

## Abstract

The treatment of environmental pollution by employing microorganisms is a promising technology, termed bioremediation, which has several advantages over the other established conventional remediation techniques. Consequently, there is an urgent inevitability to develop pragmatic techniques for bioremediation, accompanied by the potency of detoxifying soil environments completely. The bioremediation of contaminated soils has been shown to be an alternative that could be an economically viable way to restore polluted soil. The soil environments have long been extremely polluted by a number of contaminants, like agrochemicals, polyaromatic hydrocarbons, heavy metals, emerging pollutants, *etc.* In order to achieve a quick remediation overcoming several difficulties the utility of biosurfactants became an excellent advancement and that is why, nowadays, the biosurfactant mediated recovery of soil is a focus of interest to the researcher of the environmental science field specifically. This review provides an outline of the present scenario of soil bioremediation by employing a microbial biosurfactant. In addition to this, a brief account of the pollutants is highlighted along with how they contaminate the soil. Finally, we address the future outlook for bioremediation technologies that can be executed with a superior efficiency to restore a polluted area, even though its practical applicability has been cultivated tremendously over the few decades.

## Abstract 2

In this approach, a number of microorganisms that produced biologically derived surfactants are significantly utilized to break down hazardous pollutants.

## Graphical abstract

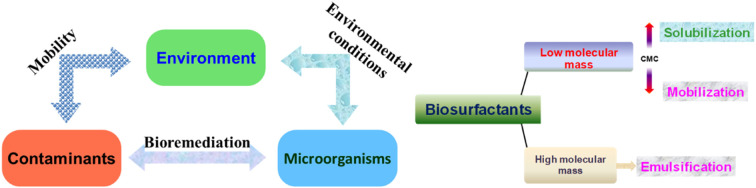

## 1. Introduction

Soil is a complex environmental component that presents as a mediator between groundwater and air; it is very difficult to regenerate. Basically, soil pollution is associated with the various pollutants exposed through human and other animal activities. The contaminating pollutants directly or indirectly penetrate into the soil environments through different channels. This leads to the concentration of the pollutants exceeding beyond the maximum soil capacity of accommodation as well as assimilation. Soil pollution readily depends on the contamination of both aspects, and includes several well-known factors, like the extensive use of pesticides and herbicides, incessant disposal of hazardous products, accidental oil spills, acid rain, and the discharge of intensely polluting agents from industries.^1,2^ The continuous growing impact of human activity and industrial development has severely influenced the environment through pollution with water insoluble organic hazardous chemicals. Since soil has a very complex structure (composed of organic matter, gases, minerals, liquids and micro-organisms), it is highly susceptible to be polluted by multifarious contaminants because of the binding ability with numerous chemical species. Nowadays industrial developments are increasing to enhance human society, the effect of which is responsible for creating an unhealthy environment. Several hazardous materials, including organic pollutants, heavy metals, solvents, plastics, nuclear hazards, and medical wastes are present in the soil with a high enough concentration to pollute the soil matrix.^3–5^ The existence of a number of hydrophobic organic hazardous components in the environment has caused serious threats, including mutagenic, carcinogenic and teratogenic effects, to mammals. Organic pollutants consist of a wide variety of organic xenobiotic chemicals, almost insoluble in the aquatic environment and that may impend in water, sediment or biota. The organic pollutants, especially gasoline, plastics, paints, pesticides, polycyclic aromatic hydrocarbons (PAHs), adhesives, polychlorinated biphenyls, benzene, ethylbenzene, toluene and many more, are actual hazards to the soil environment. Therefore, soil contamination is a serious environmental problem across the world, for which the agricultural output of land decreases, the health of human beings faces many serious threats, and underground water becomes polluted, which ultimately creates a negative impact on the ecosystem and biodiversity. Thus, it is necessitated to clean up contaminated soil to get our environment back into its native form. Several technologies have been developed to maintain the quality of soil,^6,7^ emphasizing the transformation and detoxification of pollutants. It is worth accounting for the disadvantages of physical and chemical remediation processes concerning high economic factors required by the treatment of a huge excess of hazardous waste leading to a direct detriment of the local ecosystem.^8^ The many advantages of bioremediation,^9^ such as the most economical, environmentally friendly and simplest approaches, over other existing technologies enable bioremediation for the permanent elimination of contaminants from polluted sites. Bioremediation is a smarter technology associated with the microbial species present in the environment involving the biological removal of pollutants from the contaminated soil matrix. The bioremediation process is not only concerned with the microorganisms but also deals with several metabolites which they fabricated, in particular surface-active agents. Hence, extensive studies have been reported on the remediation of contaminated soil by numerous research groups since its commencing.^10–14^ Bioremediation techniques essentially require suitable soil conditions for microbial activity and the accurate selection of the active microorganisms. The highly recalcitrant nature of some organic and inorganic pollutants makes some limitations to this promising technique. In this circumstance, biologically derived natural surfactants play a leading role to address the limitation of detoxifications using the bioremediation approach.^15^ Surface-active compounds are synthesized with the help of microorganisms, known as biosurfactants, and have the ability to assist the bioremediation process to be effective in the removal of pollutants from a soil matrix.^16–20^ The fact that biosurfactants can improve the solubilization of pollutants from contaminated soil and increase their solubility, which in turn enhances their bioavailability, is the cause of the growing interest in them.^21^ Decontamination of soil can be performed *in situ* (at the site where it is occurring – ‘soil flushing’) or *ex situ* (after the excavation of contaminated soil – ‘soil washing’), applying systematic arrangements. This includes stabilization, solidification, extraction of vapor as well as electrochemical techniques and biological or thermal treatment.^22^ It is best to first present a brief highlight of different classes of soil pollutants, possible sources of soil contamination, chemistry of biosurfactants, and microbial production of biosurfactants to illustrate the significant issues. Then the biosurfactant assisted bioremediation of soil (contaminated by hydrocarbons, heavy metals, and emerging pollutants) is demonstrated comprehensively together with numerous literature reports. Finally, a brief discussion on (a) the mechanistic action of biosurfactants on soil contaminants and (b) a comparison of biosurfactants with synthetic surfactants is essential in order to sketch out the significance of biosurfactants for soil bioremediation. At the end, a description about the limitations of biosurfactant assisted remediation along with concluding remarks make this review comprehensive.

## 2. Soil pollutants: possible route of contamination

Soils are the leading environmental sink for a variety of polluting agents, contamination of which increases the risk of ecological systems around the world. A wide diversity of inorganic and organic chemicals and pollutants may appear in soil in various identities, compositions and concentrations. Soils are polluted by the following primary chemicals and/or substances (Table 1).

**Table 1:** Numerous kinds of soil pollutants with their sources

Pollutants	Sources/contamination path	Ref.
Naphthalene, phenanthrene, pyrene	Fossil fuel combustion, traffic emissions, coke ovens, diesel and gasoline engines	35
DDT	Directly used as pesticides and agricultural pests and vectors	36
HCB	Air emissions, combustion products, municipal incinerators, volatilization and leaching from landfills	37
PCBs	Widely used as coolants and lubricants in transformers, also used in numerous electrical equipment	38
α-HCH and β-HCH	Leaching and volatilization, deposition of wastes from chemical industries	38
Heavy metals (Pb, Cd, Cu, Hg, Sn, Zn)	Disposal of wastes from industrial areas, leaded gasoline and paints, application of fertilizers and pesticides, sewage sludge, wastewater irrigation, spillage of petrochemicals, coal combustion residues	39
Bisphenol A (BPA), phthalates, parabens, chlorpyrifos	Plastics, plasticizers, pesticides	40

### 2.1 Agrochemicals

Crop production is mainly governed by the presence of the required number of micronutrients: nitrogen, phosphorous and potassium (NPK). In order to meet the rising demand for food, synthetic fertilizers have been used extensively for a long time to maximize crop output. The excessive consumption of synthetic fertilizers changes the superiority and enrichment of the soil and, as a consequence, the soil becomes polluted. Together with the use of pesticides, including insecticides, herbicides, and fungicides, these are a matter of serious concern to the world because of their better interaction with the constituents of soil.^23^ A significant number of degraded products and residues build up in the soil environment as a result of the ongoing use of agrochemicals in agriculture, which may pose serious hazards to soil and food chain pollution.^24^ The types of agrochemicals are: insecticides (*e.g.*, chlordimeform, diazinon, dimethoate, aldrin, chlordane, DDT, flumethrin, permethrin, *etc.*); acaricides (*e.g.*, fenpyroximate, menthol, formic acid, tau-fluvalinate, thymol, *etc.*); herbicides (*e.g.*, acetanilides, barban, alachlor, chlorbromuron, 2,4-D, 2,4,5-T, *etc.*); bactericides (*e.g.*, copper hydrochloride, copper oxychloride, copper sulfate, different rice blast nets, dithane, polytrin, ridomil, and many more).

### 2.2 Polycyclic aromatic hydrocarbons (PAHs)

Due to the poisonous, mutagenic, and carcinogenic properties, organic pollutants coupled with petroleum, such as PAHs, are regarded as hazardous pollutants. The PAHs are very common organic pollutants, having two or more fused aromatic rings liable to pollute the soil, and tend to retain in the soil matrix for a range of time. The number of aromatic benzene rings determines the classification of PAHs; those with two or three rings are classified as low molecular weight (LMW) PAHs, including anthracene, naphthalene and phenanthrene, while those with four or more rings are classified as high molecular weight (HMW) PAHs, including chrysene, pyrene, benzo(*a*)pyrene, coronene, *etc.* Depending on their molecular weight, they have a variety of physicochemical and toxicological properties. Typically, both anthropogenic and natural activities result in the production of these.^25^

### 2.3 Persistent organic pollutants (POPs)

The POPs are organic compounds with a greater affinity towards the soil matrix, accounted by their bioaccumulation for a long period.^26^ The lipophilic and hydrophobic properties of POPs facilitate a strong interaction with cell membranes and consequently they can readily enter into the food chain of living organism, which leads to a significant risk for human health.^27^ Examples of POPs are: polychlorinated biphenyl (PCB), polychlorinated dibenzofurans (PCDFs), polybrominated diphenyl ethers, per- and polyfluoroalkylated substances (PFAS), short-chain chlorinated paraffins, *etc.*^28^

### 2.4 Toxic heavy metals

The atmospheric deposition of heavy metals from industrial areas, the disposal and treatment of waste, the use of commercial fertilizers, the use of sewage sludge, and other processes resulting from the deterioration of various materials are examples of heavy metal inputs into the environment.^29,30^ Many heavy metals are present in soils, and average quantities vary across the globe. Examples include Cu (20 mg kg^−1^), Cd (0.06 mg kg^−1^), Cr (20–200 mg kg^−1^), Pb (10–150 mg kg^−1^), Ni (40 mg kg^−1^), and Zn (10–300 mg kg^−1^). Nevertheless, the basic parent materials of pollution can lead to 10–1000 times higher concentrations of heavy metals in metal-rich soils. Hazardous metals in soils, such as As, Cd, Cr, Cu, Hg, Pb, and Zn, are thought to be the most contaminating metals there are, and their fundamental characteristics include non-degradability, persistence, bioaccumulation, and biomagnification in a food chain.^31^

### 2.5 Radioisotopes

Environmental radionuclides can be created artificially or naturally. According to estimates, on average, 79% of the radiation to which people are exposed comes from natural sources, 19% from medical use, and the remaining 2% is caused by radiation from nuclear power plants and weapons testing.^32^ The global effects of atmospheric nuclear weapons development and the functioning of nuclear facilities, however, have caused the majority of the public's worries about radiation from radionuclides. These two activities have contaminated substantial tracts of land with radionuclides and released a significant number of man-made radionuclides into the environment. Soils have been contaminated for much longer than anticipated by normal fission product radionuclides such ^137^Cs and ^90^Sr. A long time after the initial contamination, radioactive nuclei like ^40^K, ^90^Sr, ^137^Cs, ^232^Th, and ^238^U, as well as some radioactive wastes produced by nuclear reactors, are readily bio-available in the soil profile and are absorbed by plants, making them available for further dispersion within food chains.^33^

### 2.6 Emerging pollutants

Soils are affected by long-term exposure to the following class of chemicals which can promote microbial resistance and are ultimately involved in reducing the quality and excellency of agricultural soils. In addition to that, more than a few chemicals, attributed as endocrine disrupting chemicals (EDCs), are notorious for disturbing the function of endocrine systems, which can cause cancerous tumors, birth defects, and other developmental disorders, in association with adversely affecting human reproductive systems by reducing the sperm count.^34^ Examples are: bisphenol A (BPA), phthalates, tributyltin, methyl mercury, methylparaben, atrazine, *etc.*

## 3. Chemistry of biosurfactant

Synthetic surfactants are used for widespread applications, including detergency, personal care products, organic transformation, and medicinal purposes. Still, the toxicological and non-biodegradable characters of synthetic surfactants are creating considerable headaches. Surfactants from natural sources, known as biosurfactants, emerged to be an environmentally benign alternative to their synthetic community^41,42^ due to their high biodegradability, multi-functionality, low toxicity, and environmental compatibility.^43^

### 3.1 Biosurfactant: structure, properties and origin

**Fig. 1:**
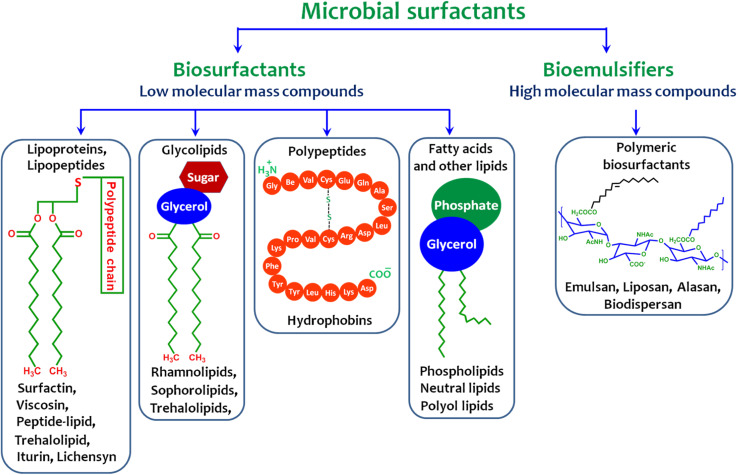
Classification of microbial surfactants along with their representative architectures.

Microbial surface-active chemicals are a class of structurally diverse molecules produced by different microorganisms. They are primarily categorized according to their chemical nature and microbial source. They are composed of an acid, peptide anions or cations, mono-, di-, or polysaccharides, which comprise their hydrophilic moiety, and fatty acids, which comprise their hydrophobic moiety. These structures have a wide range of features, such as the capacity to reduce the surface and interfacial tension of liquids and to create micelles and microemulsions between various phases. These substances can be generally classified into two classes: biosurfactants, which are low-molecular-weight (<1500 Da) substances, such as glycolipids, proteins, and lipopeptides,^44^ and bioemulsifiers, which are high-molecular-weight polymers of polysaccharides, lipoproteins, or lipopolysaccharide proteins (Fig. 1).

**Fig. 2:**
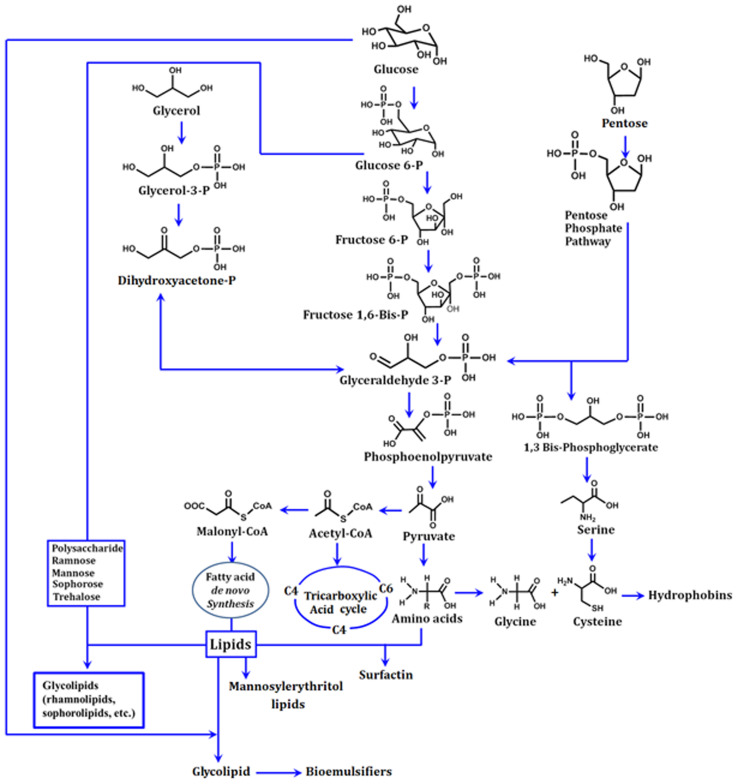
Biological synthetic routes for the synthesis of a variety of lipopeptides (*i.e.* surfactin), glycolipids (*i.e.* rhamnolipids, sophorolipids, *etc.*), bioemulsifiers and hydrophobins using carbohydrate substrates (redrawn and extended from ref. 50–52).

Glycolipids are the microbial surfactants that have been investigated the most. Fig. 2 shows the proposed biosynthetic route for the production of glycolipids. Rhamnolipids, trehalolipids, sophorolipids, and mannosylerythritol lipids (MELs) are some of the most well-known substances among these.^45^ Since carbohydrates are the only available source of carbon for the synthesis of biopolymers, the formation of the hydrophilic moiety *via* a glycolytic pathway as well as the lipogenic pathway (lipid formation) is inhibited by microbial metabolism (shown in Fig. 2). A variety of enzymes break down a water-soluble substrate like glucose to generate intermediates like glucose 6-phosphate (G6P) in the glycolytic pathway. This G6P is one of the primary precursors of carbohydrates found in the hydrophilic zone of biopolymers. Glucose is initially transformed to pyruvate by glycolysis, then pyruvate is converted into acetyl-CoA, which further combines with oxaloacetate and results in the formation of malonyl-CoA, which is subsequently converted into a lipid.

Microbial species present in the environment can be able to generate a variety of amphiphilic compounds with significant surface activities. Being a surfactant, biologically derived natural surfactants are also reported for their extensive applications in various fields.^46^ Biosurfactants, analogous to synthetic surfactants, form micelle like nano-aggregates within the concentration range of 1–200 mg L^−1^. However, a larger CMC value of biosurfactants has been accounted recently; for example, glycolipids and glycoproteins, produced by *Acinetobacter baumannii*^47^ and *Rhizopus arrhizus*,^48^ exhibit CMCs of 1200 and 1700 mg L^−1^, respectively. The rhamnolipids and surfactin are two well characterized natural surfactants that lower the surface tension of water from 73 to 30 mN m^−1^, as revealed for other biosurfactants.^49^

The applications of bio-derived surfactants are, though not completely, explored, but not limited to the industries of cosmetics, pharmaceuticals, agriculture, food, textile, painting, petroleum recovery and many others; the use of biosurfactants for remediation purposes is quite a lucrative and essential approach to remove pollutants from the environment through microbial degradation method.^53^ The contaminants, primarily organic pollutants, are usually highly recalcitrant and hardly removed by conventional remediation technologies. In addition to this, some toxic heavy metals can strongly interact with the soil matrix, which produces lots of complexities proportionally to eliminate the hazardous metals from the soil. The well-established bioremediation technique, assisted by the presence of biologically active surfactants, is a method through which the detoxification of polluted soil is executed at ease. The main problem arising in the course of a microbial bioremediation process is the availability of pollutants to the microorganism.

**Fig. 3:**
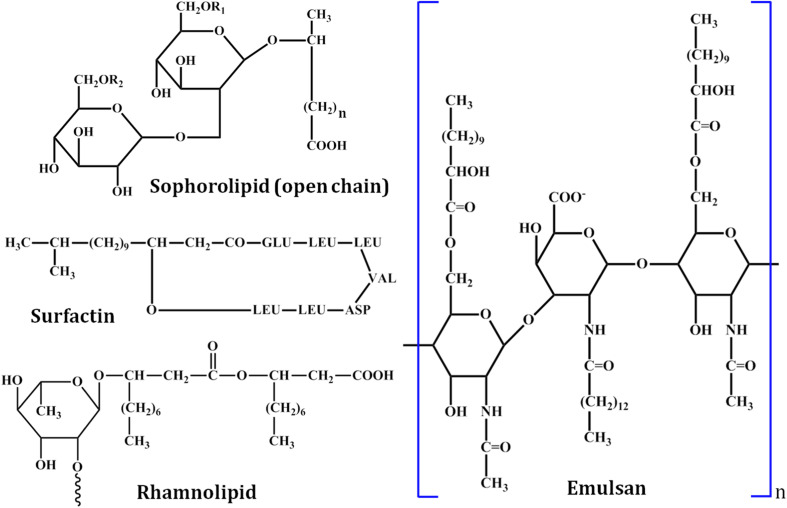
Chemical structures of selected biosurfactants: sophorolipid (open chain form), surfactin, rhamnolipid, and emulsan.

The insoluble nature of organic pollutants is susceptible to restricting their availability to the microorganisms. Biosurfactants are more effective in order to get a significant remediation of pollutants, since the bioavailability of contaminants, including hydrophobic compounds and heavy metals, are realistically increased in the presence of biosurfactants. Many surfactants of various types have already been studied for their potential use in accelerating the biodegradation of organic pollutants like PAHs.^54^ Before going into detail about the application part, it is necessary to characterize the structures of the biosurfactant (Fig. 3).

Based on their structural diversity, biosurfactants are classified into different categories, as mentioned earlier. Herein, inclusive information of the biosurfactants of various classes are summarized (Table 2) together with the microbial origins and related applications.

**Table 2:** Examples of the common biosurfactants, producing microbes and specific applications

Class	Biosurfactant	Microorganism	Application	Ref.
Lipopeptides	Surfactin	*Bacillus subtilis*, *Bacillus pumilus* A	Petroleum industry, environmental and agricultural applications	55 and 56
	Lichenysin	*Bacillus licheniformis*	Anti-adhesion activity	57 and 58
	Viscosin	*Pseudomonas fluorescens*	Antibacterial, antiviral, antitrypanosomal therapeutic	59
	Anikasin	*Pseudomonas fluorescens* HKI0770	Used as amebicide, inhibit protozoan grazing	60
	Serrawettins	*Serratia marcescens*	Used in medical, pharmaceutical, agricultural and petroleum industries	61
Glycolipids	Rhamnolipids	*Pseudomonas aeruginosa*, *Pseudomonas putida*	Applied for environmental remediation	62
	Mannosylerythritol lipids	*Pseudozyma* sp., *Candida antartica*, *Ustilago maydis*	Repair of damaged hair, moisturization of dry skin, activation of fibroblast and papilla cells and protective effects in skin cells, and as an antioxidant	63
	Trehaloselipids	*Rhodococcus* sp., *Arthrobacter* sp.	Biomedical and industrial application in addition to bioremediation	64
	Sophorolipids	*Candida bombicola*, *Candida apicola*, *Candida batistae*	Amelioration of skin physiology, skin restructuring and repair	65
	Cellobiolipids	*Ustilago maydis*	Antifungal activity	65
Phospholipids, neutral lipids, and fatty acids	Phospholipid	*Thiobacillus thiooxidans*	Agents for respiratory failure	66
	Fatty acids	*Corynebacterium lepus*	Used as emulsifiers in food industries	67
	Neutral lipids	*N. erythropolis*	Partition the organic compound into a micellar core	68
Polymeric	Biodispersan	*A. calcoaceticus*	Prevent flocculation, fracturing of limestone, used in the paint industry	69
	Emulsan	*Acinetobacter calcoaceticus*	Hydrocarbon-in-water emulsions are stabilized	70
	Mannan protein emulsifiers	*Saccharomyces cerevisiae*	Stabilizes hydrocarbon in water emulsions	71
	Alasan	*Acinetobacter radioresistens*	Hydrocarbon-in-water emulsion stabilizer	72
Siderophore	Flavolipids	*Flavobacterium* sp.	Stable emulsifier used in remediation applications	73

## 4. Biosurfactant assisted bioremediation of soil

**Fig. 4:**
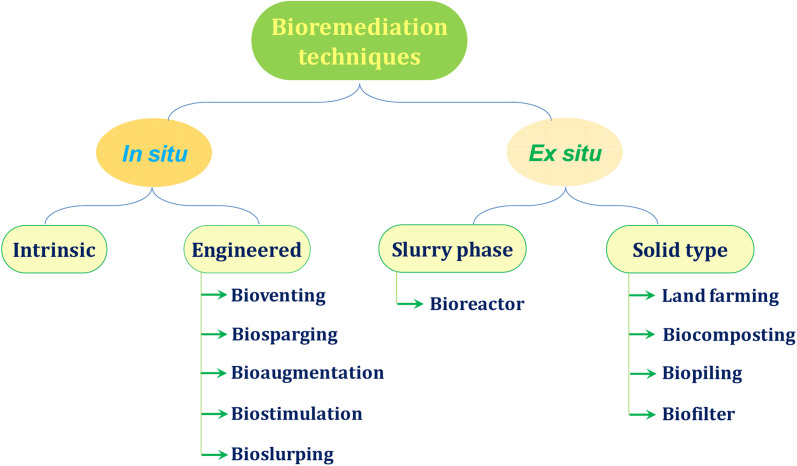
Bioremediation processes are mainly classified into two categories: *in situ* and *ex situ*. The *in situ* and *ex situ* processes have different pathways of bioremediation.

Considering the severe risk factors for human health on account of soil pollution, there is an urgency to remove the intact pollutants from the soil media. The term “remediation” interprets elimination, deterioration or transformation of harmful contaminants into less detrimental chemicals. As revealed from the literature, the bioremediation approach is one of the best techniques^74^ established so far, continually employed from laboratory research to industry for exploring the biodegradation of contaminants. Soils are contaminated with a number of pollutants from hydrophobic organic compounds to emerging pollutants, including PAHs, POPs, toxic heavy metals, radionuclides, and so many other contaminants. In order to degrade or detoxify a wide variety of harmful compounds from the soil media using bioremediation techniques, the following factors are getting the major concern: (a) perfect assortment of microorganism, (b) maintaining favorable conditions (pH, aeration, temperature, *etc.*) for the remediation, (c) bioavailability of contaminants on spatial and temporal scales, and finally (d) the nature of the contaminants itself. In general, the biosurfactant influenced bioremediation method corresponds to *in situ* and *ex situ* pathways followed by the selection of biosurfactant addition (Fig. 4). The *in situ* methods involve the on-site generation of biosurfactant (soil flushing) while the *ex situ* methods require the introduction of externally synthesized biosurfactants into the contaminated site (soil washing).^75^ The introduction of externally synthesized biosurfactants is an existing approach which satisfactorily produces an excellent removal efficiency from laboratory to large industrial scales. Numerous biosurfactants, their employment in degrading or detoxifying distinct contaminants with a potential removal efficiency are described in the following sections.

### 4.1 Biosurfactants used for the bioremediation of hydrocarbon contaminated soil

**Fig. 5:**
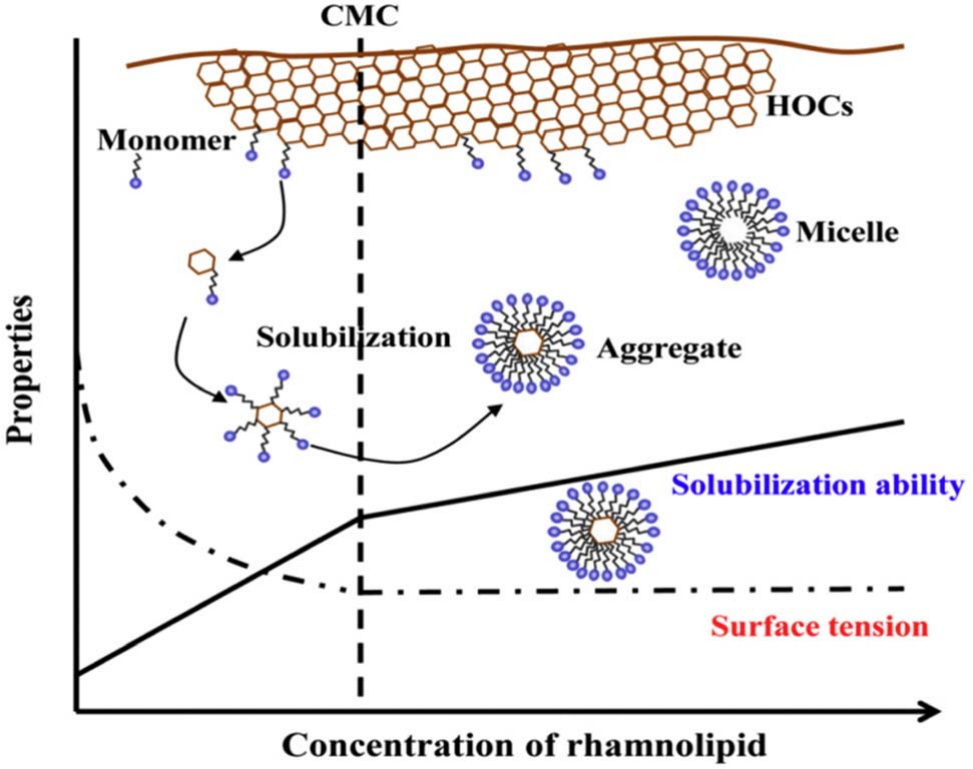
Schematic representation of rhamnolipid-enhanced aqueous dispersion of HOCs. Reprinted with permission from ref. 82 Copyright 2018 Elsevier.

The lower solubility of hydrophobic PAH molecules accounts for their strong attachment with the soil matrix and hence it is tricky to remove them from soil media. A number of microorganisms generate natural surfactants which have a vibrant role in the biodegradation of contaminated PAHs and organic hydrophobic compounds from soil. Biologically derived surfactants significantly augment the rate of PAH biodegradation by increasing the bioavailability of hydrophobic substrates. Basically, hydrophobic organic compounds are not easily accessible, hence biologically produced surfactants improve the efficiency of desorption and accessibility of hydrophobic compounds so that organic pollutants become easily bio-accessible.^76^ Several researchers extensively investigated the potential activity of widely diverse microbial species exploited for PAH degradation. The biosurfactant rhamnolipid is a promising one in the degradation of hydrocarbons, as reported elsewhere (Table 3). Noticeably, bio-trickling saponin filters are involved in removing hexane.^77^ A pseudomonas strain-containing biofilm has also been shown to work as a (bio) filter for volatile organic chemicals, breaking down hexane and generating biosurfactants. Microorganisms that generate biosurfactants from hydrocarbons are vital in biofiltering because they can eliminate the pollution and prevent biomass collection.^78^ The degradation of a petroleum hydrocarbon from contaminated soil mainly depends on the useful step, the initial washing of soil by the biosurfactant.^79^ Soil washing, a practical approach in the removal of contaminants, proceeds through two mechanistic pathways: a soil roll-up mechanism and solubilization.^80^ It was observed that the biodegradation of PAHs increased as the concentration of the biosurfactant increased up to a threshold level. After this threshold level, the degradation efficiency of the biosurfactant reduces to some extent since a higher dose of biosurfactant interferes with the cellular membrane of the microbial species. Fig. 5 represents how the concentration of rhamnolipid influences the aqueous dispersion of hydrophobic organic compounds.^81,82^ Biosurfactants not only influence the solubility of organic contaminants but also control the surface hydrophobicity of the microbial cell. Again, the surface hydrophobicity of the cell drives cell adherence to hydrophobic organic compounds and cell-to-cell interactions.^83^

**Table 3:** The biosurfactant mediated remediation of organic contaminants from soil

Organic chemicals	Microbial species and/or biosurfactant	Removal efficiency (%)	Ref.
Phenanthrene (Phen)	*Pseudomonas stutzeri*	Phen: 86–95	83
	*Bacillus simplex*		
Fluorine (F)	*Bacillus pumilus*	F: 65–86	
Pyrene	*Pseudomonas aeruginosa* SP4	84.6	84
Anthracene (A)	*Aeribacillus pallidus*	A: 92–96	85
Fluorene (F)	*Bacillus axarquiensis*	F: 83–86	
Phenanthrene (Phen)	*Bacillus siamensis*	Phen: 16–54	
Pyrene (P)	*Bacillus subtilis* subsp. *inaquosorum*	P: 51–71	
Phenanthrene (Phen)	*Aeribacillus pallidus* SL-1	Phen: 80	86
Pyrene (P)		P: 50	
*n*-Hexadecane (*n*-Hex)	*Pseudomonas aeruginosa* san ai	*n*-Hex: 80	87
*n*-Nonadecane (*n*-Dec)		*n*-Dec: 98	
Fluorene (F)		F: 96	
Phenanthrene (Phen)		Phen: 50	
Pyrene (P)		P: 41	
Pyrene	*Paenibacillus dendritiformis* CN5	83.5	88
Gasoline	*Ludwigia octovalvis* plantation and biosurfactant	96.5	89
Total petroleum hydrocarbon (TPH)	*Maize plantation* and rhamnolipid	58	90
Motor oil	*Candida sphaerica*-biosurfactant	90	91
Petroleum hydrocarbon	*Bacillus subtilis* A21-biosurfactant	64.5	92
Diesel oil	*Staphylococcus epidermidis* EVR4	84	93

**Fig. 6:**
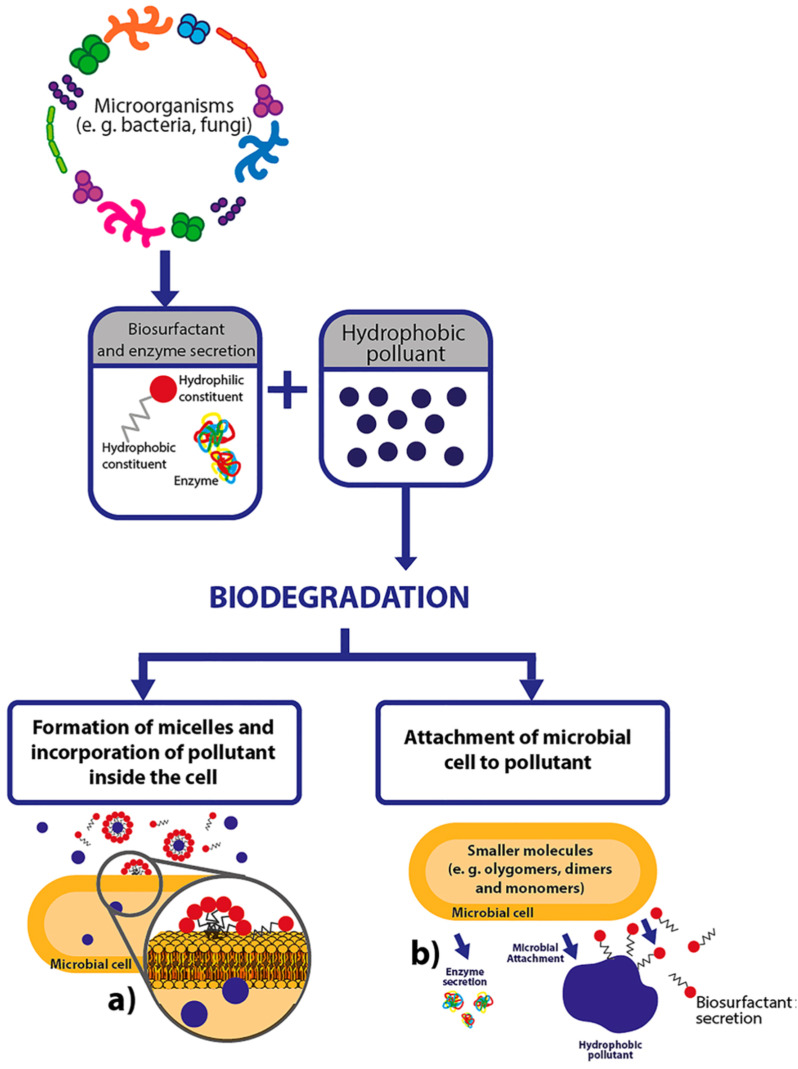
Probable mechanistic actions of biosurfactants in the microbial decay of hydrophobic pollutants from soil media: (a) generation of micelles and encapsulation of organic contaminants within the microbial cell and (b) microbial attachment of hydrophobic pollutants. Reprinted with permission from ref. 95.

In bioremediation processes of hydrophobic compounds from contaminated soil, the biosurfactants conduct three different modes of action based on their concentration and molecular weight.^94^ At lower concentrations (<CMC), the biosurfactant diminishes the surface and interfacial tension between soil–water and air–water systems. This reduction of interfacial force promotes the contact of biosurfactants with the soil-contaminant and thereby favors the mobilization of organic pollutants. When a sufficient amount of biosurfactant exists in the polluted soil matrix micellization occurs and those developed micelles can encapsulate the organic contaminant at the interior formed by the hydrophobic tail part of the biosurfactant molecules. Thus, the solubilization of hydrophobic organic compounds using the aggregated nanostructure of biosurfactants (above the CMC) is another mode of the bioremediation mechanism. These two mechanistic modes of the biosurfactant mediated remediation process are entirely based on the concentration of the employed biosurfactant having a low molecular weight (Fig. 6). When a biosurfactant of high molecular weight is used for soil bioremediation purposes, especially for the removal of hydrocarbon contaminated soil, then the emulsifying ability of the biosurfactant contributes the mechanistic role. A higher molecular weight biosurfactant encourages the biodegradation process following the solubilization and emulsification of organic hydrophobic contaminants.

In order to advance our knowledge of the mechanistic action of biosurfactants for the degradation of hydrophobic contaminants, additional research is actually needed to comprehend the actual interaction of biosurfactants with cells.^96^

### 4.2 Biosurfactants used for the bioremediation of heavy metals from soil

**Fig. 7:**
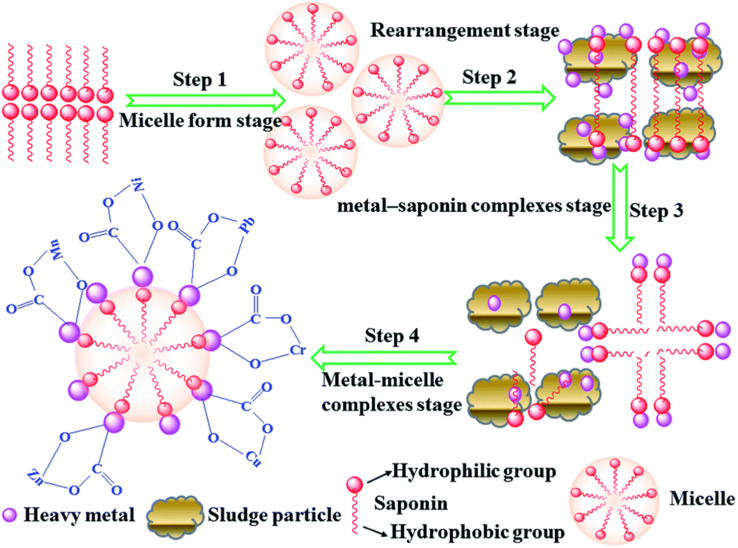
Mechanism of saponin assisted heavy metal removal from soil–sludge. Reprinted with permission from ref. 109 Copyright 2017 Royal Society of Chemistry.

**Fig. 8:**
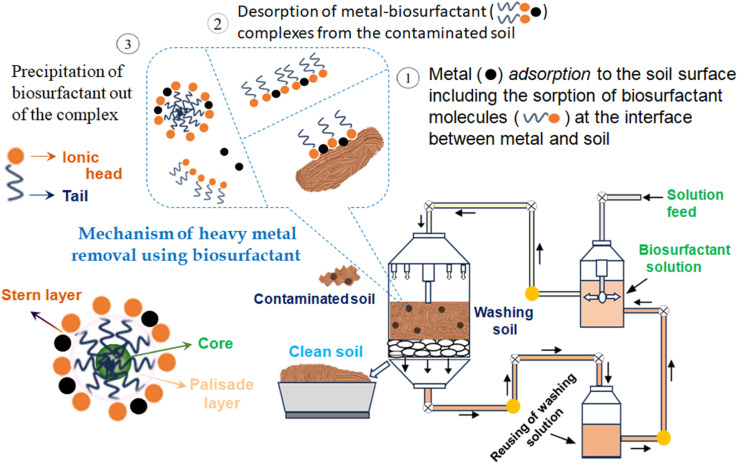
Technique for heavy metal removal from contaminated soil using biosurfactants and the obtained removal rates for Cu, Zn, Cr, and Cd of 90–100% in normal conditions.

Metals can be readily accumulated in the soil media and thereby easily enter into the soil–food chain,^97^ which becomes a matter of concern to the entire environmentalist community and all other divisions of research. Soil microorganisms have a remarkable role on remediating these metallic toxins.^98^ Phytoremediation is a comparatively less destructive, environmentally friendly and cost-efficient remediation technique which allows soil clean-up over a large scale.^99^ In this context, plant growth promoting rhizobacteria, PGPR, assisting the remediation of toxic metal contaminated soil was reported to be an efficient bioremediation technique.^100^ In another recent study, the biomineralization technique was materialized when the microbial induced phosphate precipitation (MIPP) approach^101^ was shown to be feasible in the remediation of the potentially toxic ions. *Providencia alcalifaciens* strain 2EA was significantly active to remediate lead ions from lead contaminated soil through a biomineralization process for the inorganic phosphate.^102^*Pseudomonas putida* and *Leclercia adecarboxylata* were proficient for the bioremediation of Pb^2+^ ions.^103^*Pseudomonas aeruginosa* strain BS2 produced rhamnolipid biosurfactant successfully removed 92% of Cd and 88% of Pb from artificially contaminated soil.^104^ The greater potential of biosurfactant generated from the *Candida tropicalis* yeast was accounted for by the removal of Zn and Cu from polluted sand.^105^ The plant based green surfactant saponin in association with a microorganism generated rhamnolipid biosurfactant was introduced to treat cadmium contaminated soil; the maximum uptake of cadmium was 39.06 mg kg^−1^ of the rhamnolipid surfactant used in the phytoremediation pathway.^106^ The soil washing treatment is another approach for the bioremediation of contaminated soils containing more than one heavy metal. Better results were obtained when multiple biosurfactants, having affinities towards numerous heavy metals, were applied in a soil washing process. Such a kind of advancement in the bioremediation of more than one heavy metal from polluted soils was achieved with the employment of saponin, rhamnolipid and tannic acid.^107^ Rhamnolipid-sa1 alone was reported to be effective to reduce iron up to 60.34% from the contaminated soil.^108^ Another plant extracted biosurfactant, saponin, was believed to be efficient for removing heavy metals (Cu, Zn, Cr, Pb, Ni, Mn) using a sequential sludge washing method through the proposed mechanism in Fig. 7.^109^Table 4 represents the impact of biosurfactants on the soil bioremediation of heavy metals with the corresponding removal efficiency. Biosurfactants can also stimulate heavy metals' mobilities along two separate paths—either through lowering the interfacial tension or by producing micelle like aggregates. On account of their amphiphilic nature, primarily at lower concentration, ZPC molecules adsorb at the interfaces of soil–water and metal–soil. This supports ameliorating soil wettability by minimizing the interfacial tension and strength of binding positively charged metal ions to soil materials. Subsequently, the heavy metal ions get complexed with a negatively charged biosurfactant, providing stabilization of the metal–biosurfactant conjugates. The strength of the metal–biosurfactant interaction is stronger than that of the metal–soil particle, leading to a more readily expulsion of the metal–biosurfactant complex from contaminated zones. Metal ions prefer to bind with oppositely charged particles or ions and exchange the equally charged ions (electrostatic interactions or ion exchange, eqn (1)).^110^ Ultimately, desorption of heavy metal ions takes place from the soil matrix and its complexation is noticed by the micelle like structure of the biosurfactant.^111^ A model mechanistic pathway is portrayed in Fig. 8, which represents the performance of a biosurfactant on heavy metal removal from contaminated soil.^112^

1 Soil–M^*n*+^ + R–(COOH)_*m*_ + H_2_O → R–O–M^*n*+^–(COOH)_*m*_ + 2H

**Table 4:** The biosurfactant enhances the remediation of heavy metals from contaminated soil

Heavy metals	Bioremediator species and/or biosurfactant	Removal efficiency (%)	Ref.
Cd	Rhamnolipid and saponin	ND	113
Cr, Pb, Cd, Ni, Cu	Di-rhamnolipid from *Pseudomonas aeruginosa* BS2	ND	114
Fe	*Candida sphaerica*	95	115
Zn		90	
Pb		79	
Cu	Rhamnolipid from *Pseudomonas aeruginosa* MTCC 2297	74	116
Cu	Saponin	60	109
Zn		65	
Cr		67	
Pb		40	
Ni		57	
Mn		35	
Ni	Rhamnolipid and saponin	87	117
Cr		71	
V		70	
Cd	Rhamnolipid with Citric acid	76.4	118
Pb		28.1	

It is indispensable to have much skill and experience about the origin, chemical structure and resulting properties of the biosurfactants to realize the exact mechanisms of biosurfactant activity in soil bioremediation. The mechanisms of bioremediation are connected with the complicated interactions between microorganisms, biosurfactant, pollutant and soil, as demonstrated in the following section.

### 4.3 Biosurfactants used for bioremediation of emerging pollutants from soil

Bioremediation is an emerging technology by which environmental restoration can be easily achieved following the detoxification of emerging pollutants blended with the soil matrix, as discussed in several reports^.^^119–122^ Biosurfactant plays a physiological role in increasing the bioavailability of soil bound emerging pollutants. The biosurfactant assisted removal of emerging organic contaminants occurs through two steps: mobilization and solubilization. Recently, the degradation of organophosphorus pesticides (*e.g.*, Phox) from tainted soil using a plant growth promoting rhizobacteria, *Bacillus amyloliquefaciens* YP6, was successfully investigated.^123,124^ In another study, phenanthrene and imidacloprid were significantly removed by a pesticide degrading microbe, *Bacillus thuringiensis* isolated from marine sediment.^125^ Recent investigations regarding the role of biosurfactant on the bioremediation of emerging and predominating pollutants are encapsulated in Table 5. Recently, a *Lysinibacillus sphaericus* IITR51 strain generated rhamnolipid was reported to enhance the solubility of hydrophobic pesticides and thereby increase the bioavailability of organic chemicals.^126^

**Table 5:** Biosurfactant-promoted bioremediation of soils contaminated with emerging pollutants

Pollutants	Microbial species and/or biosurfactant	Removal efficiency (%)	Ref.
Triclosan	Soil indigenous microbes and rhamnolipid	94	127
DDT	*Arthrobacter globiformis* and rhamnolipid	64.3	128
Carbendazim	*Rhodococcus* sp. D-1 and rhamnolipid	97.3	129
Endosulfan	Mixed bacterial culture	99	130
α- and β-endosulfan	Natural surfactant extracted from mesquite seed and guar gums	65–94 (for α-)	131
		41–80 (for β-)	
Diethyl phthalate	*Pseudomonas* sp. DNE-S1 produced biosurfactant	97.8	132
Atrazine	*Bacillus velezensis* MHNK1 produced surfactin	100	133
Quinalphos	*Pseudomonas aeruginosa* Q10	94	134
β-Cypermethrin	*Pseudomonas aeruginosa* CH7	90	135
Hexachlorocyclohexane	*C. striatus* plantation and *Rhodococcus erythropolis* ET54b	33	136
Epoxiconazole and fludioxonil	*Pseudomonas* sp., *Ochrobactrum* sp. and *Comamonas* sp.	56	137
Cyprodinil	*Acinetobacter* sp.	78	138

Indeed, a biosurfactant molecule can enter the cellular phospholipid membrane and alter its fluidity and permeability.

Biosurfactants assist the adsorption of pollutants by amplifying the permeability of the bacterial cell membrane through:

• An adsorption process at the outer leaflet.

• Shifting to the inner membrane.

• Insertion between the phospholipid bilayer.

The membrane fluidity of a cell is also altered by biosurfactants when the proportion of saturated to unsaturated fatty acids in the lipid bilayer diminishes. The larger the quantity of unsaturated fatty acids in the membrane, the higher is its fluidity. A better fluidity signifies an effective transportation of hydrophobic organic compounds through the membranes of a microbial cell.

**Fig. 9:**
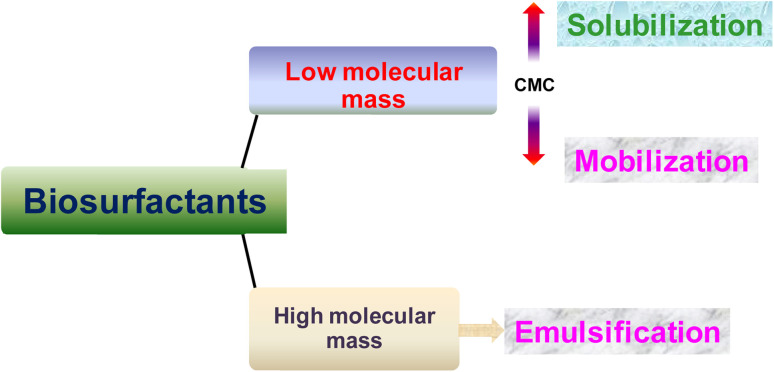
Functions of biosurfactants in the remediation of various soil pollutants.

Finally, the function of biosurfactants in soil bioremediation processes is represented in Fig. 9.^122^

## 5. Comparison of biosurfactants with synthetic surfactants for purposes of soil bioremediation

Solubilization utilizing surfactants at concentrations above their CMC values has been extensively researched in *in situ* soil washing.^139^ It is thought that nonionic surfactants, like triton, tween, and brij, which have a high hydrophobicity, are excellent for promoting the dissolution of hydrophobic organics in soil. However, it was demonstrated for nonionic surfactants that the desorption efficiency of petroleum hydrocarbons was not significantly impacted by the increase in surfactant concentration from 2× to 10× CMC.^140^ At surfactant concentrations above the CMC, the biodegradation of contaminants in soil systems has also been observed to be inhibited. Moreover, a lot of regularly used synthetic surfactants are poorly biodegradable and toxic, and their use may cause an accumulation of environmentally hazardous substances in soil.^141^ Biosurfactants made by microorganisms have recently discovered a new use in environmental clean-up procedures. In comparison to synthetic surfactants, biosurfactants have a number of distinct advantages, such as biocompatibility and biodegradability, multifunctional properties, a stable activity under extreme environmental conditions (such as high or low temperature and high pressure, pH and salinity), and can therefore be more successful in the remediation of contaminated soil.^142–144^ In earlier experimental studies, the effectiveness of synthetic and bio-surfactants in the soil bioremediation process was reported. For example:

(a) Rhamnolipid and surfactin, two biosurfactants, were compared to synthetic surfactants (Tween 80 and Triton X-100) for the removal of petroleum hydrocarbons from damaged soils. Rhamnolipids, surfactin, and synthetic surfactants like Tween 80 and Triton X-100 were all utilized at 0.2% mass each. Findings indicated that total petroleum hydrocarbons could be removed from polluted soil by 23%, 14%, 6%, and 4%, respectively. This demonstrated how much more effective biosurfactants are than synthetic surfactants.^145^

(b) SDS's capacity for removing substances was contrasted with that of biosurfactants, like saponin and rhamnolipid. The study revealed that SDS was more effective than rhamnolipid and saponin at removing crude oil from the soil. Different surfactants, however, have varying degrees of affinity for crude oil's constituents. For instance, saponin and SDS interact well with aromatic hydrocarbons while SDS is better at removing aliphatic hydrocarbons. SDS is more expensive and less biodegradable than biosurfactants but is more efficient.^146^

(c) In contrast to Triton X-100 (synthetic surfactant), which is non-biodegradable under anaerobic conditions and only partially biodegradable under aerobic conditions (soluble COD removal efficiency of 47.1% after 10 days at concentrations below 900 mg L^−1^), rhamnolipid biosurfactants are biodegradable under both aerobic and anaerobic conditions (soluble COD removal efficiency of 74% after 10 days and 47.2% after 6 days, respectively).^147^

Considering the benefit of microbial surfactants, including easy synthesis from renewable feed stocks, biodegradability, low toxicity, and higher foaming ability, over those of the synthetic surfactants suggests a high demand in a variety of industries. So biosurfactants appeared as the best candidate to replace the preexisting commercially available synthetic surfactants. However, there are various challenges in the synthesis, isolation, purification, characterization and use of them, especially at the commercial level. Extraction of green surfactants from plants and microbial species is a very costly and time-consuming process, since a mixture of numerous species are present together.^148^ Thus, a few critical limits of biosurfactants need to be revealed in context of soil bioremediation for the generation of healthy future.

## 6. Critical limits and future prospect of biosurfactants

Biosurfactant assisted technology is not very appropriate for the high scale remediation of organic contaminants; indeed, biosurfactants produce a synergistic effect with the native microbial community. Two major critical boundaries of biosurfactants are required to be resolved before establishing a practical application. Preliminary, the production and isolation of biosurfactants by maximum strains is reasonably small and needs to be highly elevated. Normally, the development conditions of bacteria are optimized. In this regard, it has been reported that *Bacillus amyloliquefaciens* A3 exhibits a satisfactory yield of biosurfactants. The mutant 1–24 of *Bacillus amyloliquefaciens* A3 supplied an important direction to the bioremediation of petroleum hydrocarbon-based contaminants from a practical standpoint. Bioremediation of *in situ* contaminated soil by *Bacillus amyloliquefaciens* A3 follows through ARTP mutagenesis.^149^

However, some methods, such as engineering bacterial growth and bacterial mutagenesis, are mostly applied to further progress. These bacteria are prepared for the bioremediation of petroleum-based hydrocarbons. The second-most issue that needs to be settled requires a special analysis which accounts for the evaluation of the potential efficiency by applying biosurfactants to decontaminate petroleum hydrocarbon contaminated soils.

## 7. Conclusion

Soil is the most fundamental but complex component that secures food safety and human health. It retains a key function in protecting the environment to safeguard and sustain the ecological balance. Soil health restoration is a great issue since soil pollution is a hidden danger below our feet. So, needless to say that now an environmentally friendly remediation technology is important for society. One of the recent symposiums (GSOP18), held in 2018 with a theme song “It is time to fight soil pollution: be the solution to soil pollution,” was addressed for a consciousness about the urgency of the conservation of soils. Bioremediation is comparatively a smarter tool to fight soil pollutants since it deals with the biological treatment of contaminants. In this approach, a number of microorganisms that produce biologically derived surfactants are significantly utilized to break down hazardous organic materials. Microbial species increase the rate of the biodegradation process through mobilization and solubilization of contaminants, resulting in a substantial detoxification of soil. Biosurfactant-enhanced soil washing is a well-recognized permanent treatment for soils contaminated with PAHs, heavy metals, and other organic compounds.

Soil bioremediation technologies can be executed more effectively by the following approach:

(a) Mixed culture media can be more adjuvant to soil polluted with multiple hazardous substances.

(b) Biosurfactants derived from plant together with microbial species can be more efficacious than employing them separately for the bioremediation of mixed contaminants.

(c) Integrated treatment can be another way of remediation and reclamation of land contaminated with hazardous materials.

(d) Requirement to advance biosurfactant based detoxification and biodegradation techniques through the exploitation of genetically engineered microbes for extremely contaminated sites.

The cost and availability of these compounds, however, pose the biggest challenge to the employment of biosurfactants in the bioremediation of soil. Therefore, more cost-effective, eco-friendly technologies are unquestionably necessary to address the present issue, since the contaminants' type and extent are always changing, creating new problems and raising a permanent risk to the entire ecosystem. On a practical level, there is a significant distinction between the laboratory setting and the actual remediation of oil-contaminated soil site restoration. In fact, it is exceedingly challenging for laboratory results of remediation to be manifested and implemented into the actual remediation field. Nevertheless, on account of the complexity issues of the reality provisos, the diversity in the type of different pollutants, the unequal distribution of contaminants and the variation of soil pollution extent, an individual remediation technique cannot indulge all types of contaminated land.

## Author contributions

BS contributed to the conceptualization and supervision. PS contributed to the writing – original draft. AG and SK contributed to the writing – review and editing. All authors contributed to the article and approved the submitted version.

## Conflicts of interest

The authors declare no competing financial interests.

## Supplementary Material
